# Association between Edinburgh Postnatal Depression Scale and Serum Levels of Ketone Bodies and Vitamin D, Thyroid Function, and Iron Metabolism

**DOI:** 10.3390/nu15030768

**Published:** 2023-02-02

**Authors:** Kiwamu Noshiro, Takeshi Umazume, Mayumi Inubashiri, Megumi Tamura, Masayoshi Hosaka, Hidemichi Watari

**Affiliations:** 1 Department of Obstetrics and Gynecology, Hokkaido University Graduate School of Medicine, Sapporo 060-8638, Japan; 2 Fukuzumi Obstetrics and Gynecology Hospital, Sapporo 062-0043, Japan

**Keywords:** ketone body, 3-hydroxybutyric acid, pregnancy, postpartum depression, Edinburgh Postnatal Depression Scale

## Abstract

Suicide due to postpartum depression is the most common perinatal-related death and is a social concern in Japan. Nutritional deficiencies during pregnancy may contribute to postpartum depression; therefore, we investigated the relationship between postpartum depression and nutritional status during pregnancy and postpartum. We focused specifically on ketone bodies because they are known to protect brain cells. The relationship between the Edinburgh Postnatal Depression Scale (EPDS) scores and the serum levels of ketone bodies and vitamin D, thyroid function, and iron metabolism was examined. Overall, 126 pregnant women were identified for the study, and 99 were eventually included in the analysis. We defined an EPDS score of ≥9 as being positive for postpartum depression, and serum ketone levels were found to be higher in the group with an EPDS score of ≥9 during the second trimester; however, there were no other significant findings. We may be able to predict postpartum depression from a pregnant woman’s serum ketone levels in the second trimester. There was a positive correlation between the EPDS scores at 3 days and 1 month postpartum (r = 0.534, *p* < 0.001). EPDS scores assessed in the early postpartum period may be useful for the timely detection of postpartum depression.

## 1. Introduction

Suicide is the most common cause of perinatal death in Japan. According to the data from the Tokyo Medical Examiner’s Office, the number of suicides among pregnant and postpartum women from 2015 to 2020 was 25 in total or 5.4 per 100,000 live births in Tokyo [1]. Postpartum depression is a major concern as more women attempt suicide after giving birth [2]. Additionally, the incidence of all types of psychiatric disorders 0 to 90 days postpartum was 11.3 per 1000 person-years as compared with 3.8 per 1000 person-years among non-pregnant women [3]. Since a systematic review has shown a negative relationship between postpartum depression and prenatal attachment, even if postpartum depression does not always lead to suicide, poor maternal-fetal bonding is a serious problem [4]. A lack of maternal–fetal bonding is more likely to lead to child abuse, and the increasing number of child abuse cases has also become a serious problem in recent years in Japan [5]. It is suggested that the Edinburgh Postnatal Depression Scale (EPDS) could be an effective predictor of suicidal ideation [6] and postpartum depression [7]. If it is proven that one of the causes of elevated EPDS scores is nutritional status during pregnancy and postpartum, early diagnosis and nutritional therapy may be possible.

In patients with severe morning sickness, ketone bodies are produced by adipose tissue and detected on urinalysis. Our previous study showed that serum total ketone body levels during the third trimester of pregnancy were higher than those during the first trimester [8]. We initially thought that the serum ketone body levels would be high during the first trimester (when fatty acid metabolism becomes dominant due to eating disorders caused by morning sickness) and then gradually decrease toward the third trimester; rather, we found that they peaked in the third trimester. This fact suggests that an increase in serum ketone body levels during pregnancy is an active change and may have some physiological benefits. We hypothesized that the increase in the levels of 3-hydroxybutyric acid, a ketone body, during the third trimester could possibly have a positive effect on the maternal brain and prevent postpartum depression; thus, the higher the levels of 3-hydroxybuteric acid, the lower the EPDS score. 3-hydroxybutyric acid has been shown to be beneficial for the central nervous system and can affect many metabolic processes, which are associated with aging and apoptosis. It blocks the NLR family pyrin domain containing 3 (NLRP3) inflammasome and attenuates caspase-1 and IL-1β secretion in mouse models; additionally, it is believed to reduce hypoglycemia-related neuronal apoptosis, increase the number of motor neurons, increase neuronal activity and angiogenesis, and protect neuronal cell cultures from the development of amyloid pathology [9,10,11]. A study on a rodent model has also shown that 3-hydroxybutyric acid administration reduces the levels of hippocampal tumor necrosis factor-α, which is tightly regulated by the NLRP3 inflammasome [12]. A clinical benefit of ketone bodies is that fasting and elevated ketone levels improve the symptoms of patients with major depressive disorder who did not sufficiently respond to initial antidepressant drug treatment [13]. Furthermore, 3-hydroxybutyric acid acts protectively on brain cells and slows the progression of Alzheimer’s disease and Parkinson’s disease [14]. Notably, a ketogenic diet may reduce the frequency of epileptic seizures [15,16].

We also assessed the levels of 25-hydroxyvitamin D (25[OH]D), thyroid function, and the presence of anemia within the study cohort, all of which have been identified as risk factors for postpartum depression in previous studies [17,18,19]. It is known that vitamin D acts through nuclear receptors to control the expression of different genes and exhibits strong anti-inflammatory and neuroprotective effects [20]. Iron deficiency affects the hippocampus, corpus striatum, and certain neurotransmitters, subsequently causing depression [21]. This study aimed to examine whether there is an association between the EPDS scores and serum levels of ketone bodies and vitamin D, thyroid function, and iron metabolism among pregnant women to predict postpartum depression. Moreover, this study aimed to investigate the relationship between EPDS and maternal–fetal bonding.

## 2. Materials and Methods

This study was approved by the Institutional Review Board of Hokkaido University Hospital (019-0391). The Declaration of Helsinki has been followed in this study. All participants provided written informed consent before their participation in the study. This study enrolled 126 pregnant women who were scheduled to give birth at the Fukuzumi Obstetrics and Gynecology Hospital between January and June 2021. 

We selected participants based on the following inclusion criteria: (1) Those aged ≥20 years; (2) those scheduled to give birth at the participating institution; (3) those who provided consent to participate in the study; (4) those who are Japanese; and (5) pregnant women who delivered after 36 weeks of gestation. The exclusion criteria were as follows: (1) pregnant women with depression before pregnancy; (2) those with twin pregnancy; (3) those who delivered via cesarean section; (4) those who were transferred to other hospitals; (5) those that voluntarily moved to other hospitals; and (6) those who failed to provide a blood sample. We excluded the cesarean-section group from the analysis because it was reported that the cesarean-section group was more prone to postpartum depression than the vaginal delivery group [22]. We used the EPDS to investigate if women had a trend toward postpartum depression. The EPDS has 10 questions about the state of mind, scaled up to 30 points. The higher the score, the more a participant tends to have postpartum depression. We defined an EPDS score of ≥9 as being positive for postpartum depression and that of <9 as being negative, the same as the Japanese standard [7]. We also used the maternal–fetal bonding score, which has 10 questions to assess how much women love their children, on a scale of 30 points. It was developed by Kumar and has shown good validity as a screening measure in Japan [23,24].

Blood samples were collected, and the serum levels of ketone bodies (total ketones, 3-hydroxybutyric acid, and acetoacetic acid) were measured during the second trimester (24–27 gestational weeks), third trimester (33–35 gestational weeks), at 1 day postpartum, and at 1 month postpartum. The levels of thyroid hormones (thyroid-stimulating hormone (TSH) and thyroxine), 25-hydroxyvitamin D (including vitamin D2 and D3), ferritin, and iron and total iron binding capacity (TIBC) were measured for the same time periods, except for postpartum day 1. In addition, the participant’s age, height, pre-pregnancy weight, pre-pregnancy body mass index (BMI), weight at delivery, BMI at delivery, pregnancy and delivery history, weeks of delivery, delivery pattern, 3-day postpartum EPDS and maternal-fetal bonding scores, and 1-month postpartum EPDS and maternal-fetal bonding scores were extracted from the medical records. Patients were given the EPDS and maternal–fetal bonding questionnaires in person, which were collected by the medical staff and scored. The association between the levels of serum ketone bodies, thyroid hormones, vitamin D, ferritin, and iron, TIBC, EPDS score, and maternal-fetal bonding was examined. 

### 2.1. Biochemical Tests

Serum was stored at −80 ℃ until assays were conducted for the assessment of the following three hematological factors: Total ketone bodies, 3-hydroxybutyric acid, and acetoacetic acid. These were measured using enzyme-linked immunosorbent assay kits TKB-L (KAINOS, Tokyo, Japan) and 3HB-L (KAINOS, Tokyo, Japan). Ferritin and iron levels and unsaturated iron binding capacity were measured using ARCHITECT ferritin (Abbott Japan, Chiba, Japan), Quick Auto Neo Fe (Shino-Test, Tokyo, Japan), and Quick Auto Neo UIBC (Shino-Test, Tokyo, Japan), respectively. TSH and thyroxine levels were measured using ARCHITECT TSH and FT4 abbott (Abbott Japan, Chiba, Japan). Vitamin D levels were measured using vitamin D total II (Roche Diagnostics K.K., Tokyo, Japan). 

### 2.2. Statistical Analysis

Statistical analyses were performed using the JMP Pro16© statistical software package (SAS, Cary, NC, USA). The *t*-test was used for comparison between the groups (EPDS positive vs. negative group; EPDS score increased by >3 points vs. decreased by >3 points group). Changes in variables within a group were compared using the Tukey–Kramer method with Bonferroni’s correction. Single regression analysis was used to investigate the relationship between the EPDS and hematological data (ketone bodies, vitamin D, TSH, thyroxine, ferritin, iron, and TIBC). In all analyses, statistical significance was set at a *p*-value of <0.05. A correlation of r ≥ 0.3 and r ≤ −0.3 was considered moderately significant.

## 3. Results

A total of 126 pregnant women were identified for the study. Of these, the following were excluded: 13 women who delivered by cesarean section, 7 who were transferred to other hospitals, 2 who voluntarily moved to other hospitals, and 5 whose blood samples were not available. Consequently, a total of 99 women were included in the final analysis dataset.

### 3.1. Demographic Characteristics

Among the 99 participants, 53 were primipara pregnant women. The mean (±standard deviation) age and gestational period at delivery were 30.3 ± 3.9 years and 39.3 ± 0.8 gestational weeks, respectively. The mean pre-pregnancy weight and pre-pregnancy BMI were 53.1 ± 6.9 kg and 21.0 ± 2.4 kg/m^2^, respectively. The blood samples were obtained at 26.4 ± 0.7 gestational weeks for the second trimester and at 34.8 ± 0.5 gestational weeks for the third trimester. Blood samples were collected from every participant on postpartum day 1. A 1-month postpartum checkup was conducted on day 31.9 ± 3.6. The mean EPDS scores at 3 days and 1 month postpartum were 3.39 ± 3.1 and 2.85 ± 3.0, respectively; the corresponding mean maternal-fetal bonding scores were 1.40 ± 1.6 and 0.70 ± 1.0, respectively (Table 1).

### 3.2. Blood Parameters during Pregnancy

The median 3-hydroxybutyric acid levels were 15.3 µmol/L, 42.0 µmol/L, 18.2 µmol/L, and 30.7 µmol/L in the second trimester, in the third trimester, at 1 day postpartum, and at 1 month postpartum, respectively. The median 3-hydroxybutyric acid concentration at 1 month postpartum was higher than that at 1 day postpartum (*p* < 0.001). The median TSH levels were 0.815 µIU/mL, 1.02, µIU/mL, and 0.872 µIU/mL, and the median thyroxine levels were 0.78 ng/dL, 0.88, ng/dL, and 0.93 ng/dL in the second trimester, in the third trimester, and at 1 month postpartum, respectively. At 1 month postpartum, the thyroxine levels were higher than in the third trimester (*p* < 0.001). The median vitamin D levels were 9.6 ng/mL, 8.7 ng/mL, and 11.9 ng/mL in the second trimester, in the third trimester, and at 1 month postpartum, respectively. The highest peak of the median vitamin D level was at 1 month postpartum (Table 2).

### 3.3. Association of Edinburgh Postnatal Depression Scale Scores in Different Periods and Its Relation with Maternal-fetal Bonding Scores

**Figure 1:**
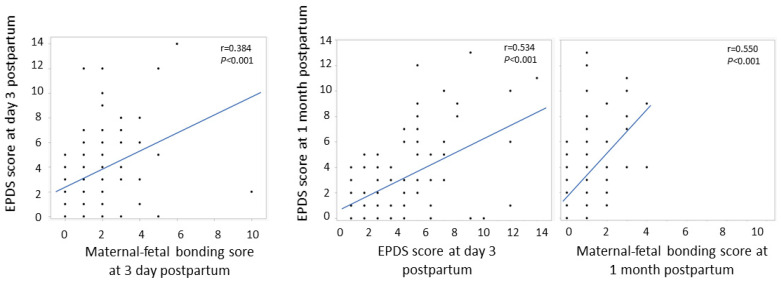
Association of Edinburgh Postnatal Depression Scale scores in different periods and its relationship with maternal-fetal bonding scores.

There was a positive correlation between the EPDS and maternal–fetal bonding scores, with a correlation coefficient of 0.384 (*p* < 0.001) at 3 days postpartum and 0.550 (*p* < 0.001) at 1 month postpartum. There was also a positive correlation between the EPDS scores at 3 days and those at 1 month postpartum, and the correlation coefficient was 0.534 (*p* < 0.001) (Figure 1).

### 3.4. Association between Edinburgh Postnatal Depression Scale Scores and Serum Levels of Ketone Bodies

In Japan, an EPDS score of >9 is defined as being positive for postpartum depression, and 7 (7.1%) women were positive at 1 month postpartum in this study. Ketone body (total ketones, 3-hydroxybutyric acid, and acetoacetic acid) concentrations from the second trimester to those at 1 month postpartum were compared between the group with an EPDS score of ≥9 and that with an EPDS score of <9. Total ketone, 3-hydroxybutyric acid, and acetoacetic acid levels were higher in the EPDS-high group during the second trimester (*p* < 0.001), although there was no difference during the third trimester, at 1 day postpartum, and at 1 month postpartum (Table 3). 3-hydroxybutyric acid levels and EPDS scores were positively correlated only in the second trimester (r = 0.442, *p* < 0.001 at 3 days postpartum; r = 0.367, *p* < 0.001 at 1 month postpartum), but not at any other time (Figure S1).

### 3.5. Association between Edinburgh Postnatal Depression Scale Score and Other Hematological Factors

In addition to ketone bodies, TSH, thyroxine, vitamin D, ferritin, iron, and TIBC were analyzed for associations with the 1-month postpartum EPDS score. There was no significant difference between the groups with EPDS scores of ≥9 and <9, and there was no correlation with the 1-month postpartum EPDS scores (Table 4 and Table S1). TSH, thyroxine, vitamin D, ferritin, iron, and TIBC were also analyzed for associations with the 3-day postpartum EPDS score, although there was no significant difference between the groups with EPDS scores of ≥9 and <9 (data were not shown).

### 3.6. Comparison of the EPDS Score Increased and Decreased Groups from 3 Days to 1 Month Postpartum 

We focused on women whose EPDS scores changed significantly from 3 days to 1 month postpartum. The comparison was made between the group in which the EPDS scores increased by >3 points from 3 days to 1 month postpartum and the group in which it decreased by >3 points over the same time period (Table 5). There was no significant difference between the two groups.

## 4. Discussion

Herein, we aimed to examine whether there was an association between the EPDS scores and ketone bodies in pregnant women during pregnancy and postpartum. We report the following findings: (1) Serum levels of 3-hydroxybutyric acid increased from the second to the third trimester and decreased after delivery. However, it rose again at 1 month postpartum; (2) there was no correlation between the EPDS scores and levels of ketone bodies or other hematological factors other than the levels of ketone bodies during the second trimester; and (3) there was a positive correlation between the EPDS and maternal–fetal bonding scores at 3 days and 1 month postpartum.

Our previous study showed that the serum levels of ketone bodies gradually increased during pregnancy [8]. As this study was conducted on postpartum depression, we focused on the period from the second trimester of pregnancy to 1 month postpartum. The increase in serum levels of ketone bodies from the second to the third trimester was consistent with that observed in a previous study [8]. The participants of this study were women with uncomplicated pregnancies; further, consistent with previous studies, our study suggested that increased ketone body levels in the third trimester could be a physiological change in normal pregnancies. After delivery, at 1 month postpartum, the ketone body levels were significantly higher than those at 1 day postpartum (Table 2).

The average EPDS score was 3.39 at 3 days postpartum and 2.85 at 1 month postpartum (*p* = 0.217). There was no significant difference in the EPDS scores at 3 days and 1 month postpartum in this study, although some studies have shown that the EPDS scores gradually improved postpartum [25,26]. If 3-hydroxybutyric acid has a protective effect against the development of postpartum depression, there may be some relationship between an increase in 3-hydroxybutyrate levels and a decrease in the EPDS scores at 1 month postpartum. However, we found no correlation between 3-hydroxybutyric acid levels and the EPDS scores other than that during the second trimester, and the relationship was opposite to that stated in the hypothesis (Figure S1).

In this study, we assumed that the higher the level of serum 3-hydroxybutyrate, which is said to have a protective effect on brain cells, the lower the EPDS score and thus the less likely the occurrence of postpartum depression [27]. This is because 3-hydroxybutyrate has been shown to have antioxidant effects that protect brain cells, and 3-hydroxybutyrate has been shown to have an advantage against diseases such as Alzheimer’s, Parkinson’s, Huntington’s, and epilepsy [14,15,16,28]. N-3 fatty acids, which have antioxidant effects similar to those of 3 hydroxybutyrate, are also said to prevent postpartum depression, and ketosis is said to improve depression [29,30]. However, this study found no relationship between the EPDS scores and serum 3-hydroxybutyrate levels other than that during the second trimester. The relationship was opposite to the hypothesis stating that higher 3-hydroxybutyrate levels are less likely to cause postpartum depression and thus was rejected. Additionally, we focused on changes in the EPDS scores from 3 days to 1-month postpartum, although there was still no significant difference between the EPDS score increased by >3 points group and decreased by >3 points group (Table 5). This result suggested that nutritional status did not improve or worsen the EPDS score.

Several previous reports suggest that vitamin D deficiency is associated with postpartum depression [31,32,33]; however, this was not evident in our study results. As the study period coincided with the coronavirus disease 2019 pandemic, all study participants must have refrained from going out, and as the major source of vitamin D is sun exposure [34], this may explain the lack of difference in the vitamin D levels among the groups with high and low EPDS scores in this study. Although there are studies that report that anemia and hypothyroidism are risk factors for postpartum depression [18,19,29], we found no relationship between these hematological factors and the EPDS scores. It may be difficult to predict postpartum depression from the results of hematological factors only because factors such as age, multiparity, social support, past history, and family background have an influence on postpartum depression [35].

In this study, all women were scored for the EPDS and maternal–fetal bonding at 1 day and 1 month postpartum. There was a positive correlation between the EPDS and maternal–fetal bonding scores, suggesting that successfully developing a bond with the infant may not be possible in those who were more prone to postpartum depression. There was a positive correlation between the EPDS scores at 3 days and 1 month postpartum (Figure 1), suggesting that using the EPDS at early postpartum would be helpful to detect postpartum depression early. 

A previous study reported a positive correlation between the EPDS scores at discharge and those at 1 month postpartum [25] and suggested that it was important to obtain the EPDS scores in the early postpartum period to identify postpartum depression. Moreover, another study calculated the EPDS scores during pregnancy and examined the tendency for depression during pregnancy [36,37]. Although it is not a common practice to investigate the EPDS scores during pregnancy in Japan, it may be beneficial to introduce EPDS testing during pregnancy for the early prediction of postpartum depression; subsequently, we may be able to find a relation with nutrition.

This study had some limitations. First, the timing of using the EPDS scores was at 3 days and at 1 month postpartum. It is common to use EPDS for the detection of postpartum depression [31,38,39]. However, some studies suggest that it is more appropriate to perform an EPDS assessment at 2–6 months postpartum than at 1 month postpartum [40,41], as in this study. There was no correlation between the EPDS scores and serum ketone body levels other than that during the second trimester, which may have been due to the inappropriate timing of the use of the EPDS. Second, the use of the EPDS for screening postpartum depression might not have been a good choice. There are several tests for screening postpartum depression, such as the “Postpartum Depression Screening Scale, “Patient Health Questionnaire 9”, and “Beck Depression Inventory [42]”, which we might have considered using. 

## 5. Conclusions

Elevated ketone levels in the second trimester may be an early indicator of postpartum depression. Although we focused on the relationship between the EPDS and nutritional status, the relationship between those who truly experience postpartum depression and nutritional status remains unclear, and further study is needed. Additionally, there was a positive correlation between the EPDS scores at 3 days and 1 month postpartum, which indicated that the use of the EPDS immediately after delivery will lead to the early detection of postpartum depression. Because there was a positive correlation between the EPDS scores and bonding scores, the EPDS may be an effective predictor of perinatal attachment, and we should conduct the EPDS a few days after delivery for both mothers and children.

## Figures and Tables

**Table 1:** Participant characteristics (*n* = 99 pregnant women).

Nulliparous Women	53 (53%)
Age, years	30.3 (±3.9)
Height, m	1.59 (±0.05)
Pre-pregnancy weight, kg	53.1 (±6.9)
Pre-pregnancy body mass index, kg/m^2^	21.0 (±2.4)
Weight gain in pregnancy, kg	10.6 (±3.5)
Gestational period at delivery, week	39.3 (±0.8)
No. of preterm deliveries before 36th gestational week	1 (1.0%)
Infant sex	
Male	50 (51%)
Weight of baby at birth, kg	3.1 (±0.3)
Height of baby at birth, cm	48.7 (±1.6)
Head circumference of baby at birth, cm	32.3 (±1.4)
Chest circumference of baby at birth, cm	31.5 (±1.4)
Time at which tests were conducted	
In second trimester, week	26.4 (±0.7)
In third trimester, week	34.8 (±0.5)
On the day after delivery, day	1 (±0)
One month checkup post delivery, days	31.9 (±3.6)
EPDS score at day 3	3.39 (±3.1)
EPDS score at month 1	2.85 (±3.0)
Maternal-fetal bonding score at day 3	1.40 (±1.6)
Maternal-fetal bonding score at 1 month post delivery	0.70 (±1.0)

Data are presented as the mean (±standard deviation) or as the number (percentage) as indicated. EPDS, Edinburgh postnatal depression scale.

**Table 2:** Hematological factors of 99 pregnant women included in the study.

	Second Trimester	Third Trimester	1 Day Postpartum	1 Month Postpartum	*p*-Value
Hematological Factors					
Total ketones, µmol/L	33.4 [17.9–210]	75.6 [32.1–367]	33.2 [14.2–75.1]	48.0 [23.4–221]	<0.001
3-hydroxybutyric acid, µmol/L	15.3 [6.3–138]	42.0 [15.3–265]	18.2 [9.0–45.5]	30.7 [14.0–143]	<0.001
Acetoacetic acid, µmol/L	16.8 [8.8–72.2]	30.0 [14.8–95.3]	14.2 [4.1– 32.2]	18.3 [6.8–68.8]	<0.001
Thyroid stimulating hormone, µIU/mL	0.815 [0.112–2.180]	1.02 [0.134–2.29]		0.872 [0.206–2.25]	0.12
Thyroxine, ng/dL	0.78 [0.67–0.95]	0.88 [0.75–1.06]		0.93 [0.82–1.09]	<0.001
Vitamin D, ng/mL	9.6 [6.4–18.3]	8.7 [5–16.5]		11.9 [8.3–20.7]	<0.001
Ferritin, ng/mL	11.8 [5.5–43.3]	10.2 [5–26.9]		26.5 [9.2–97.3]	<0.001
Iron, µg/dL	76 [29–150]	52 [26–138]		96 [45–165]	<0.001
Total iron binding capacity, µg/dL	450 [374–563]	526 [446–670]		380 [300–461]	<0.001

Hematological values are presented as the median [5th–95th percentile]. We used analysis of variance to confirm a difference in the value of the timing of the blood test.

**Table 3:** Association between EPDS scores at 1 month postpartum and ketone bodies.

	Group with EPDS Scores of ≥9	Group with EPDS Scores <9	*p*-Value
Clinical data			
Number of women	7	92	
Age, years	29.7 (2.8)	30.4 (4.0)	0.679
Height, m	1.62 (0.05)	1.59 (0.05)	0.12
Pre-pregnancy body weight, kg	54.1 (3.6)	53.0 (7.1)	0.687
Pre-pregnancy body mass index, kg/m^2^	20.7 (2.1)	21.0 (2.4)	0.757
Body weight at delivery, kg	63.5 (4.2)	63.8 (7.8)	0.934
Body mass index at delivery, kg/m^2^	24.4 (2.7)	25.3 (2.7)	0.373
Gestational age at delivery, week	39.0 (1.1)	39.3 (0.8)	0.431
Weight of baby at birth, kg	47.9 (0.7)	48.8 (1.6)	0.154
Height of baby at birth, cm	3.00 (0.23)	3.09 (0.32)	0.435
Head circumference of baby at birth, cm	32.0 (1.3)	32.4 (1.4)	0.515
Chest circumference of baby at birth, cm	30.8 (1.3)	31.6 (1.4)	0.159
Hematological factors during the second trimester			
Values calculated during the second trimester, week	26.6 [0.6]	26.4 [0.7]	0.512
Total ketones, µmol/L	67.5 [29.2–953]	32.7 [11.5- 354]	<0.001
3-hydroxybutyric acid, µmol/L	39.5 [15.3−683]	14.5 [5.4–230]	<0.001
Acetoacetic acid, µmol/L	29.0 [13.9−270]	16.5 [4.7–124]	<0.001
Hematological factors during the third trimester			
Values calculated during the third trimester, week	34.7 [0.57]	34.8 [0.48]	0.791
Total ketones, µmol/L	80.1 [49.7–367]	74.0 [16.5–482]	0.287
3-hydroxybutyric acid, µmol/L	51.3 [23.6–292]	40.0 [8.4–370]	0.249
Acetoacetic acid, µmol/L	28.8 [16.1–84.6]	30.1 [7.2–115]	0.469
Hematological factors at postpartum day 1			
Total ketones, µmol/L	26.8 [15.0–44.3]	33.5 [11.7–165]	0.258
3-hydroxybutyric acid, µmol/L	15.3 [10.8–31.7]	19.0 [8.1–121]	0.425
Acetoacetic acid, µmol/L	12.2 [3.3–16]	15.1 [2.1–44.8]	0.159
Hematological factors at 1 month postpartum			
The day of one month checkup, day	31.3 [4.1]	32.0 [3.6]	0.627
Total ketones, µmol/L	36.9 [16.7–79.9]	49.1 [19.2–606]	0.222
3-hydroxybutyric acid, µmol/L	24.4 [9.6–59.6]	30.8 [11.0–505]	0.27
Acetoacetic acid, µmol/L	13.4 [7.1–20.3]	4.2 [19.2–101]	0.126

Clinical data are presented as the mean (±standard deviation). Hematological values are presented as the median [range]. EPDS, Edinburgh postnatal depression scale.

**Table 4:** Association between EPDS scores at 1 month postpartum and hematological factors.

	**Group with EPDS Scores of ≥9**	**Group with EPDS Scores of <9**	***p*-Value**
Hematological values during the second trimester			
Thyroid stimulating hormone, µIU/mL	0.75 [0.32–1.38]	0.83 [0.01–3.02]	0.369
Thyroxine, ng/dL	0.86 [0.7–0.87]	0.78 [0.62–1.39]	0.228
Vitamin D, ng/mL	10.3 [8.9–21.1]	9.5 [4.2–19.5]	0.185
Ferritin, ng/mL	13.1 [9.0–19.4]	11.7 [5.0–21.7]	0.61
Iron, µg/dL	83 [35–105]	75 [21–210]	0.89
Total iron binding capacity, µg/dL	427 [361–571]	451 [332–576]	0.939
Hematological values during the third trimester			
Thyroid stimulating hormone, µIU/mL	0.52 [0.35–1.42]	1.02 [0.01–2.78]	0.135
Thyroxine, ng/dL	0.89 [0.76–1.06]	0.88 [0.69–1.29]	0.374
Vitamin D, ng/mL	9.2 [5.7–13.9]	8.7 [3.6–18.6]	0.909
Ferritin, ng/mL	10.6 [8.5–16.9]	9.9 [4.3–66.5]	0.711
Iron, µg/dL	79 [35–138]	52 [17–168]	0.275
Total iron binding capacity, µg/dL	512 [426–695]	527 [400–691]	0.469
Hematological factors at 1 month postpartum			
Thyroid stimulating hormone, µIU/mL	0.78 [0.21–1.2]	0.892 [0.01–3.2]	0.228
Thyroxine, ng/dL	0.95 [0.87–1.04]	0.93 [0.73–1.25]	0.415
Vitamin D, ng/mL	12.2 [10.4–14.5]	11.9 [7.2–23.7]	0.843
Ferritin, ng/mL	21 [8.4–23.6]	32 [5.8–138]	0.064
Iron, µg/dL	86 [47–136]	96 [25–198]	0.672
Total iron binding capacity, µg/dL	400 [282–477]	378 [283–488]	0.431

Hematological values are presented as the median [range]. EPDS, Edinburgh postnatal depression scale.

**Table 5:** Comparison of the EPDS score increased and decreased groups from 3 days to 1 month postpartum.

	EPDS Score Increased by >3 Points	EPDS Score Decreased by >3 Points	*p*-Value
Clinical data			
Number of women	15	21	
Age, years	30.0 (3.1)	28.9 (2.4)	0.218
Height, m	1.58 (0.04)	1.59 (0.06)	0.801
Pre-pregnancy body weight, kg	53.3 (4.3)	51.1 (6.4)	0.255
Pre-pregnancy body mass index, kg/m^2^	21.3 (1.4)	20.3 (1.9)	0.085
Body weight at delivery, kg	63.3 (3.6)	62.0 (7.9)	0.549
Body mass index at delivery, kg/m^2^	25.3 (1.3)	24.6 (2.3)	0.273
Gestational age at delivery, week	39.1 (0.6)	39.3 (0.8)	0.526
Hematological factors during the second trimester			
Second trimester, week	26.4 [0.6]	26.4 [0.7]	0.866
Total ketones, µmol/L	37.7 [22.2–123]	41.7 [14.9–354]	0.327
3-hydroxybutyric acid, µmol/L	18.1 [6.3 –85.1]	16.8 [6.1–230]	0.386
Acetoacetic acid, µmol/L	16.3 [11.8 –37.6]	21.2 [8.7–124]	0.237
Thyroid stimulating hormone, µIU/mL	0.79 [0.05–3.02]	0.76 [0.11–2.19]	0.54
Thyroxine, ng/dL	0.82 [0.74–0.98]	0.77 [0.65–0.95]	0.22
Vitamin D, ng/mL	11.0 [6.7–21.1]	10.6 [6.3–19.5]	0.532
Ferritin, ng/mL	13.1 [5.8–31.1]	12.2 [5.5–52.3]	0.412
Iron, µg/dL	74 [23–140]	81 [21–138]	0.831
Total iron binding capacity, µg/dL	431 [361–571]	475 [332–566]	0.307
Hematological factors during the third trimester			
Third trimester, week	34.6 [0.4]	34.9 [0.5]	0.09
Total ketones, µmol/L	86.8 [34.0–267]	64.9 [32.1–375]	0.669
3-hydroxybutyric acid, µmol/L	54.8 [19.2–193]	42.6 [17.0–265]	0.74
Acetoacetic acid, µmol/L	30.2 [14.9–84.6]	24.8 [7.2–110]	0.518
Thyroid stimulating hormone, µIU/mL	1.17 [0.01–2.78]	1.06 [0.26–2.22]	0.698
Thyroxine, ng/dL	0.89 [0.73–1.14]	0.89 [0.69–1.13]	0.751
Vitamin D, ng/mL	8.9 [3.6–16.5]	8.8 [5.0–17.4]	0.646
Ferritin, ng/mL	10.6 [6.1–34.1]	10.8 [6.2–41.6]	0.575
Iron, µg/dL	47 [28–121]	60 [17–138]	0.275
Total iron binding capacity, µg/dL	508 [426–695]	540 [414–691]	0.493
Hematological factors at 1 day postpartum			
Total ketones, µmol/L	29.2 [12.1–46.1]	29.3 [17.9–50.5]	0.882
3-hydroxybutyric acid, µmol/L	15.7 [8.3–26.3]	17.1 [8.1–32.1]	0.586
Acetoacetic acid, µmol/L	12.5 [3.8–24.6]	12.1 [4.6–28.9]	0.713
Hematological factors at 1 month postpartum			
The day after delivery, day	31.5 [3.5]	31.5 [3.5]	0.962
Total ketones, µmol/L	48.0 [16.7–7193]	49.0 [23.2–606]	0.336
3-hydroxybutyric acid, µmol/L	30.9 [9.6–107]	28.3 [12.1–505]	0.275
Acetoacetic acid, µmol/L	20.4 [7.1–86.1]	19.4 [5.5–101]	0.781
Thyroid stimulating hormone, µIU/mL	1.11 [0.01–1.9]	0.76 [0.21–2.65]	0.673
Thyroxine, ng/dL	0.93 [0.84–1.25]	0.96 [0.83–1.17]	0.767
Vitamin D, ng/mL	11.5 [7.6–16.5]	11.1 [7.9–23.7]	0.978
Ferritin, ng/mL	23.1 [9.2–75.6]	25.2 [7.7–116]	0.629
Iron, µg/dL	105 [47–198]	102 [34–188]	0.338
Total iron binding capacity, µg/dL	368 [282–477]	380 [331–488]	0.345

Clinical data are presented as the mean (SD). Hematological values are presented as the median [range]. EPDS, Edinburgh postnatal depression scale.

## Data Availability

The data presented in this study are available upon request from the corresponding author. The data are not publicly available as it includes patient’s personal details and medical reports.

## Supplementary Materials

The following supporting information can be downloaded at: https://www.mdpi.com/article/10.3390/nu15030768/s1, Figure S1: Relationship between Edinburgh Postnatal Depression Scale (EPDS) and log transformed 3-hydroxybutyric acid; Table S1: Correlation between Edinburgh Postnatal Depression scale score and hematological factors.
