# Supplementary material for: Association between Edinburgh Postnatal Depression Scale and Serum Levels of Ketone Bodies and Vitamin D, Thyroid Function, and Iron Metabolism
Source: Nutrients. 2023 Feb 2;15(3):768. doi: 10.3390/nu15030768 (PMC9920872; doi:10.3390/nu15030768)
Supplement: Supplementary file 1 [file nutrients-15-00768-s001.zip › nutrients-2117111-Supplementary-figure.pptx]

## Slide 1
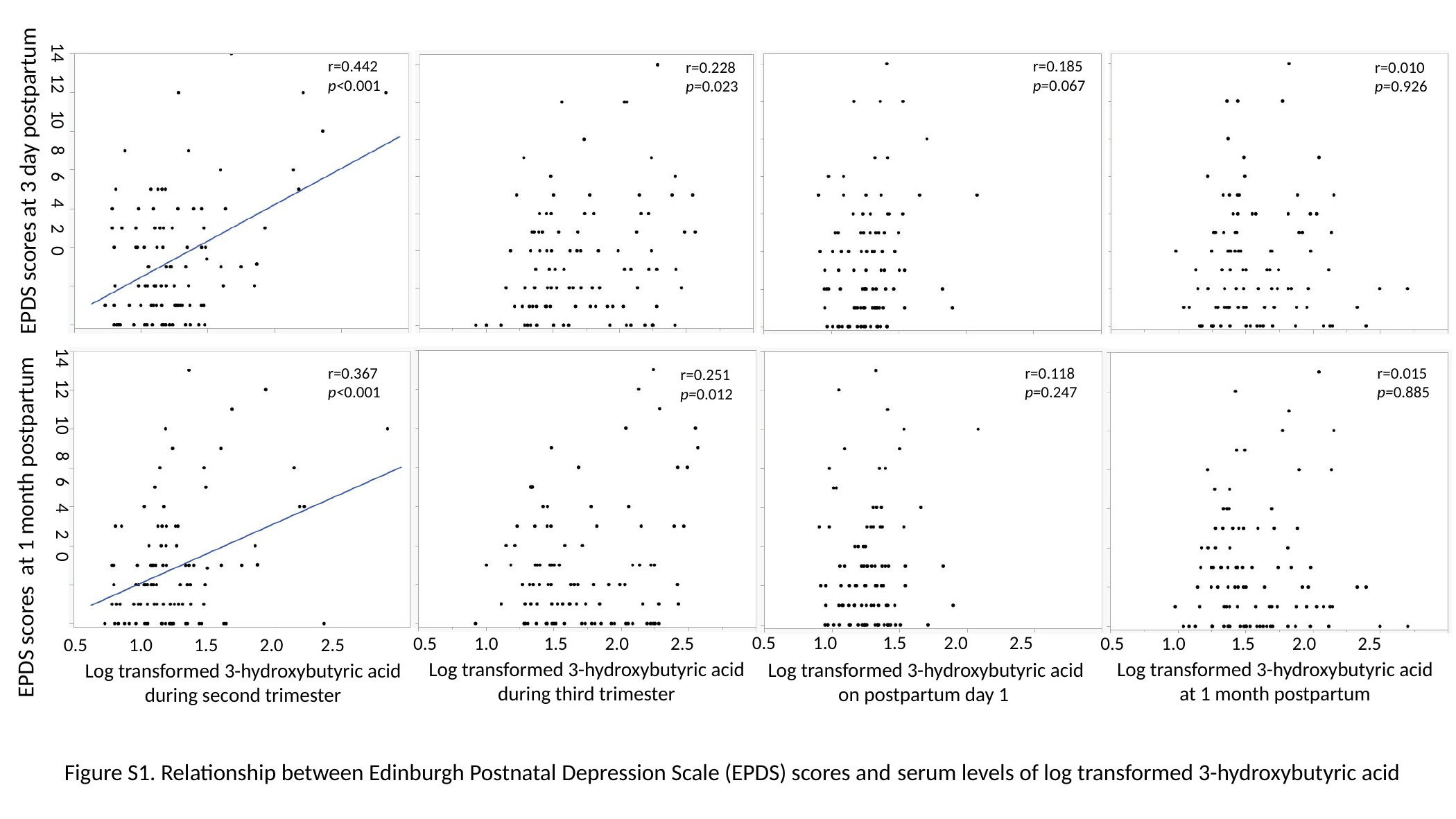

14 12 10 8 6 4 2 0
r=0.185
p=0.067
r=0.442
p<0.001
r=0.228
p=0.023
r=0.010
p=0.926
EPDS scores at 3 day postpartum
14 12 10 8 6 4 2 0
r=0.015
p=0.885
r=0.367
p<0.001
r=0.118
p=0.247
r=0.251
p=0.012
EPDS scores at 1 month postpartum
0.5 1.0 1.5 2.0 2.5
0.5 1.0 1.5 2.0 2.5
0.5 1.0 1.5 2.0 2.5
0.5 1.0 1.5 2.0 2.5
Log transformed 3-hydroxybutyric acid
at 1 month postpartum
Log transformed 3-hydroxybutyric acid
during third trimester
Log transformed 3-hydroxybutyric acid
on postpartum day 1
Log transformed 3-hydroxybutyric acid
during second trimester
Figure S1. Relationship between Edinburgh Postnatal Depression Scale (EPDS) scores and serum levels of log transformed 3-hydroxybutyric acid
