# Supplementary material for: Association between Edinburgh Postnatal Depression Scale and Serum Levels of Ketone Bodies and Vitamin D, Thyroid Function, and Iron Metabolism
Source: Nutrients. 2023 Feb 2;15(3):768. doi: 10.3390/nu15030768 (PMC9920872; doi:10.3390/nu15030768)
Supplement: Supplementary file 1 [file nutrients-15-00768-s001.zip › nutrients-2117111-Supplementary-table.pdf]

Table S1. Correlation between Edinburgh Postnatal Depression scale score and hematological factors

	EPDS at 3 day	*p*-value	EPDS at 1 month	*p*-value
**Hematological factors during second trimester**				
Total ketones, µmol/L	0.478	<0.001	0.300	0.003
3-hydroxybutyric acid, µmol/L	0.437	<0.001	0.310	0.002
Acetoacetic acid, µmol/L	0.431	<0.001	0.268	0.007
Thyroid stimulating hormone, µIU/mL	-0.170	0.093	-0.023	0.824
Thyroxine, ng/dL	-0.045	0.656	0.087	0.395
Vitamin D, ng/mL	0.102	0.318	0.112	0.272
Ferritin, ng/mL	-0.021	0.834	-0.085	0.405
Iron, µg/dL	-0.119	0.241	-0.055	0.587
Total iron binding capacity, µg/dL	0.018	0.865	-0.071	0.487
**Hematological factors during third trimester**				
Total ketones, µmol/L	0.203	0.044	0.331	<0.001
3-hydroxybutyric acid, µmol/L	0.202	0.045	0.338	<0.001
Acetoacetic acid, µmol/L	0.198	0.050	0.291	0.004
Thyroid stimulating hormone, µIU/mL	-0.020	0.845	0.090	0.378
Thyroxine, ng/dL	0.213	0.034	0.152	0.133
Vitamin D, ng/mL	0.088	0.385	0.164	0.105
Ferritin, ng/mL	0.063	0.536	-0.004	0.966
Iron, µg/dL	0.142	0.162	0.180	0.074
Total iron binding capacity, µg/dL	-0.099	0.328	-0..85	0.405
**Hematological factors at postpartum day 1**				
Total ketones, µmol/L	0.163	0.108	0.169	0.095
3-hydroxybutyric acid, µmol/L	0.173	0.087	0.171	0.091
Acetoacetic acid, µmol/L	0.104	0.305	0.123	0.225
**Hematological factors at 1 month postpartum**				
Total ketones, µmol/L	-0.052	0.607	-0.042	0.678
3-hydroxybutyric acid, µmol/L	-0.071	0.486	-0.064	0.527
Acetoacetic acid, µmol/L	0.027	0.793	0.050	0.622
Thyroid stimulating hormone, µIU/mL	-0.130	0.211	-0.011	0.919
Thyroxine, ng/dL	0.105	0.299	-0.081	0.428
Vitamin D, ng/mL	0.020	0.841	0.246	0.014
Ferritin, ng/mL	0.029	0.774	0.161	0.111
Iron, µg/dL	0.028	0.103	-0.103	0.312
Total iron binding capacity, µg/dL	-0.089	0.379	-0.059	0.564
